# The Activation of ROS/NF-*κ*B/MMP-9 Pathway Promotes Calcium-Induced Kidney Crystal Deposition

**DOI:** 10.1155/2021/8836355

**Published:** 2021-06-08

**Authors:** Yue Wu, Jiaqiao Zhang, Cong Li, Henglong Hu, Baolong Qin, Tao Wang, Yuchao Lu, Shaogang Wang

**Affiliations:** ^1^Department of Urology, Tongji Hospital, Tongji Medical College, Huazhong University of Science and Technology, Wuhan, 430030 Hubei, China; ^2^Institute of Urology, Tongji Hospital, Tongji Medical College, Huazhong University of Science and Technology, Wuhan, 430030 Hubei, China

## Abstract

Idiopathic hypercalciuria is an important risk factor for the formation of calcium-containing kidney stones. Matrix metalloproteinase-9 (MMP-9) is closely related to cell and tissue remodeling and is involved in ectopic tissue calcification. However, little is known about its role in kidney stone formation. In this study, we found that the expression of MMP-9 and that of osteoblastic-related proteins was increased in normal rat kidney epithelial-like (NRK-52E) cells following treatment with a high concentration of calcium, while the knockout or overexpression of MMP-9 could, respectively, significantly inhibit or upregulate the expression of osteoblastic-related proteins and calcium crystal deposition. In addition, apoptosis and calcium crystal deposition were significantly reduced in Sprague–Dawley rats with 1,25(OH)_2_D_3_-induced hypercalciuria following MMP-9 inhibitor I treatment. Furthermore, inhibiting reactive oxygen species (ROS) production or the nuclear factor kappa-light-chain-enhancer of activated B cell (NF-*κ*B) pathway significantly reduced calcium-induced MMP-9 expression and calcium crystal deposition. In summary, our results suggested that a high calcium concentration promotes epithelial–osteoblastic transformation and calcium crystal deposition in renal tubule cells by regulating the ROS/NF-*κ*B/MMP-9 axis and identified a novel role for MMP-9 in regulating calcium-induced calcium crystal deposition in renal tubules.

## 1. Introduction

Kidney stone disease is a common disorder of the urinary system, with an annual incidence of 114–720 cases per 100,000 adults globally, and is estimated to be rising in almost every country [1]. Moreover, approximately 50% of patients relapse within 5–10 years after the first kidney stone episode, which represents a significant health and financial burden [2]. The mechanism of kidney stone formation is complex and has never been stopped. Randall's plaque hypothesis, one of several theories of kidney stone formation, stipulates that heterotopic calcified foci composed of hydroxyapatite in the interstitium of the renal papilla is the starting point for the growth and attachment of calculi [3]. Although a variety of proteins associated with inflammation, oxidative stress, and osteoblastic transformation have been reported to be involved in the pathology of stone formation [4–6], the specific mechanism underlying the role of Randall's plaques in kidney stone formation remains unclear, and preventing the formation and recurrence of kidney stones remains an important challenge.

Nuclear factor kappa-light-chain-enhancer of activated B cell (NF-*κ*B) comprises a family of nuclear transcription factors with roles in inflammatory and immune responses and includes RelB (cRel), NF-*κ*B1 (p50, p105), RelA (p65), and NF-*κ*B2 (p52, p100). Activated NF-*κ*B binds to DNA and regulates the expression of numerous target genes [7]. Tozawa et al. [8] found that calcium oxalate crystals can activate the NF-*κ*B signaling pathway and promote osteopontin (OPN) expression in renal tubular epithelial cells. Similarly, increased reactive oxygen species (ROS) production has also been reported to activate NF-*κ*B signaling, which, in turn, activates the expression of several NF-*κ*B target genes [9]. A microarray study of genetic hypercalciuric stone-forming (GHS) rats, using Sprague–Dawley (SD) rats as controls, showed that the NF-*κ*B signaling pathway and its downstream regulatory molecules were key components of the core gene regulatory network involved in the pathophysiology of hypercalciuria in GHS rats [10].

Matrix metalloproteinase-9 (MMP-9) is a member of the zinc-dependent endopeptidase family and plays an important role in tissue remodeling, especially in the bone and the vascular system [11]. Zhang et al. [12] found that functional polymorphism in the regulatory region of the MMP-9 gene was correlated with the severity of coronary atherosclerosis, while Yasmin et al. [13] reported that circulating MMP-9 levels were associated with atherosclerosis in the large arteries of humans. Subsequent studies also proposed that MMP-9 may serve as a predictor of atherosclerosis in patients with type 2 diabetes [14, 15]. These results suggested that MMP-9 may be involved in the pathology of ectopic calcification. The kidney stone disease is an ectopic calcification disorder, and 80% of kidney stones are calcium-containing [16]. Most patients present with idiopathic hypercalciuria characterized by normal serum Ca^2+^ levels and increased urinary calcium excretion. We previously reported that MMP-9 expression was significantly increased in the kidneys of GHS rats and that MMP-9 is a downstream target of the NF-*κ*B signaling pathway [10]. These results implied that MMP-9 may play an important role in kidney stone formation.

Here, we focused on the role of MMP-9 in kidney stone formation in a high-calcium microenvironment and explored the underlying mechanism and associated regulatory network. We found that a high calcium concentration stimulated calcium salt deposition in renal tubular epithelial cells and promoted the expression of MMP-9, whereas silencing MMP-9 expression elicited the opposite effect. The results of the present study further contribute to elucidating the mechanism involved in kidney stone formation.

## 2. Materials and Methods

### 2.1. Reagents and Antibodies

Dulbecco's modified Eagle's medium (DMEM) and phosphate-buffered saline (PBS) were purchased from Hyclone (Logan, UT, USA). Fetal bovine serum (FBS) was purchased from Gibco (Grand Island, NY, USA). Anhydrous calcium chloride powder (Cat# C4901), the Annexin V-FITC apoptosis detection kit (Cat# A9210), Alizarin Red S (ARS) (Cat# A5533), dimethyl sulfoxide (DMSO) (Cat# D2650), the oxalic acid colorimetric assay kit (Cat# MAK179), the calcium colorimetric assay kit (Cat# MAK022), and bovine serum albumin (BSA) (Cat# V900933) were purchased from Sigma–Aldrich (St. Louis, MO, USA). N-Acetylcysteine (NAC) (Cat# HY-B0215), BAY 11-7082 (Cat# HY-13453), and cell counting kit-8 (CCK-8) (Cat# HY-K0301) were purchased from MedChemExpress (NJ, USA). 2′,7′-Dichlorofluorescein diacetate (DCFH-DA) (Cat# 50101ES01) and the terminal deoxynucleotidyl transferase-mediated dUTP-biotin nick end-labeling assay (TUNEL) apoptosis detection kit (Cat# 40308ES20) were purchased from Yeasen (Shanghai, China). TRIzol (Cat# R0016), radioimmunoprecipitation assay (RIPA) (Cat# P0013B), paraformaldehyde (4%) (Cat# P0099), and the lactate dehydrogenase (LDH) cytotoxicity assay kit (Cat# C0016) were purchased from Beyotime Institute of Biotechnology (Jiangsu, China). The PrimeScript RT Reagent Kit (Perfect Real Time) (Cat# RR037A) and TB Green Premix Ex Taq II (Tli RNaseH Plus) (Cat# RR820A) were purchased from TaKaRa (Japan). 1,25(OH)_2_D_3_ (Cat# ab141456) was purchased from Abcam (Cambridge, MA, USA). MMP-9 inhibitor I (Cat# 444278) was purchased from Merck Millipore (Billerica, MA, USA). The Rat MMP-9 ELISA Kit (Cat# E-EL-R0624c) was purchased from Elabscience (Wuhan, China). Primary antibodies against MMP-9 (Cat# ab76003), OPN (Cat# ab8448), E-cadherin (ab231303), and beta-actin (Cat# ab8227) were purchased from Abcam. Antibodies against NF-*κ*B p65 (Cat# 8242), phospho-NF-*κ*B p65 (Cat# 3033), I*κ*B*α* (Cat# 4814), phospho-I*κ*B*α* (Cat# 2859), and runt-related transcription factor 2 (RUNX2) (Cat# 12556); and horseradish peroxidase-labeled anti-rabbit (Cat# 7074) and anti-mouse (Cat# 7076) antibodies were purchased from Cell Signaling Technology (Beverly, MA, USA).

### 2.2. Cell Culture and Treatment

NRK-52E cells were purchased from the Type Culture Collection of the Chinese Academy of Sciences (Shanghai, China) and cultured in DMEM containing 10% FBS in a humidified atmosphere with 5% CO_2_ at 37°C. The medium was renewed every 2–3 days. The experiments were carried out on passage 3–5 cells.

Anhydrous calcium chloride powder was used to configure different Ca^2+^ concentrations to treat the NRK-52E cells for 24 h. Cells were also pretreated for 2 h with or without ROS scavenging agent NAC and the NF-*κ*B pathway inhibitor BAY 11-7082, followed by treatment with or without Ca^2+^ (0.7 mg/ml).

### 2.3. Cell Viability and Apoptosis Assays

A CCK-8 assay was used to detect cell proliferation. Briefly, cells were seeded in 96-well plates at a density of 2 × 10^3^ cells/well. CCK-8 (10%) was added to the wells followed by incubation at 37°C for 3 h. The absorbance was then measured at 450 nm using a spectrophotometer (Thermo Fisher Scientific, MA, USA).

The Annexin V-FITC apoptosis detection kit and flow cytometry (CytoFLEX, Beckman Coulter, USA) were used to analyze apoptosis levels in NRK-52E cells treated with different Ca^2+^ concentrations. After 24 h of calcium treatment, the cells were washed three times with precooled PBS and then incubated and stained with Annexin V-FITC and propidium iodide (PI) at room temperature for 15 min in the dark. Finally, the stained cells were analyzed by flow cytometry.

### 2.4. Intracellular ROS Measurement

Intracellular ROS levels were detected using the DCFH-DA fluorescent probe. NRK-52E cells were treated with a high concentration of Ca^2+^ for 24 h and then incubated with DCFH-DA at 37°C for 30 min in the dark. The cells were then washed three times in serum-free medium, and intracellular ROS levels were detected by flow cytometry (CytoFLEX, Beckman Coulter).

### 2.5. LDH Cytotoxicity Measurement

The LDH assay kit was used to evaluate toxicity-induced membrane damage in NRK-52E cells following the manufacturer's protocol. Briefly, NRK-52E cells were seeded in 96-well plates at a density of 2000 cells/well and incubated at 37°C overnight. After 24 h of treatment with different calcium concentrations (0, 0.7, or 1.0 mg/ml), 50 *μ*l of cell suspensions from each group (control and the different calcium treatment groups) were transferred to a new 96-well plate. Stop solution (50 *μ*l) was added to each well, followed by 100 *μ*l of freshly prepared reaction mixture, and incubation at 15–25°C for 30 min. The absorbance of each sample at 490 nm was determined using a spectrophotometer (Thermo Fisher Scientific).

### 2.6. Immunofluorescence Analysis

After treatment, the NRK-52E cells were fixed in 4% paraformaldehyde for 15 min and permeabilized with 0.5% Triton X-100 in PBS at room temperature for 20 min. After blocking with goat serum at room temperature for 30 min, the cells were incubated at 4°C overnight with antibodies targeting MMP-9 (1 : 300) and NF-*κ*B-p65 (1 : 400). The next day, the cells were incubated with fluorescent secondary antibody at 20–37°C for 1 h and counterstained with DAPI for 5 min. Finally, the samples were observed and the images were captured under a fluorescence microscope (BX53, Olympus, Tokyo, Japan).

### 2.7. Transmission Electron Microscopy (TEM)

NRK-52E cells were collected and fixed in 2.5% glutaraldehyde for 2–4 h. The cells were then immobilized with 1% osmium acid for 2 h, dehydrated using a graded alcohol series, permeated overnight with acetone, and stained with uranium and lead. Finally, the samples were observed and the images were captured under a HT7800 TEM (Hitachi, Japan).

### 2.8. Cell Transfection and Real-Time Quantitative Polymerase Chain Reaction

An MMP-9 expression plasmid and small interfering RNA (siRNA) targeting MMP-9 (MMP-9 siRNA) were used for the gain- and loss-of-function analysis, respectively. The MMP-9 expression plasmid and its negative control (NC) plasmid were synthesized by GeneChem (Shanghai, China). MMP-9 siRNA was synthesized by RiboBio (Guangzhou, China). The sequences of the siRNAs used in this study are 5′-GCGCUGGGCUUAGAUCAUUTT -3′ (MMP-9 siRNA) and 5′-UUCUCCGAACGUGUCACGUTT-3′ (NC siRNA). NRK-52E cells were seeded in a 6-well plate at 5 × 10^5^ cells per well and transfected according to the manufacturer's instructions (Invitrogen).

Total RNA was extracted using TRIzol reagent and reverse-transcribed into cDNA using the PrimeScript RT Reagent Kit (Perfect Real Time). Real-time quantitative polymerase chain reaction was performed using the TB Green Premix Ex Taq II (Tli RNaseH Plus) in an ABI Prism 7300 system (Thermo Fisher Scientific). Beta-actin was used as the reference gene, and the 2^-∆∆Ct^ method was used to calculate the fold change of the target genes. The sequences of all the primers used in this study are shown in Supplementary Table S1.

### 2.9. Western Blot Analysis

NRK-52E cells and rat tissues were lysed with RIPA buffer containing protease inhibitors to obtain total proteins. The prepared proteins were separated by 10% sodium dodecyl sulfate-polyacrylamide gel electrophoresis (SDS–PAGE) and transferred to polyvinylidene difluoride (PVDF) membranes (Millipore). After blocking with 5% BSA for 2 h, the membranes were incubated overnight at 4°C with primary antibodies targeting MMP-9 (1 : 500), OPN (1 : 500), RUNX22 (1 : 1000), E-cadherin (1 : 1000), p65 (1 : 1000), p-p65 (1 : 1000), I*κ*B*α* (1 : 1000), p-I*κ*B*α* (1 : 1000), and *β*-actin (1 : 200). Then, the membranes were washed with TBST and incubated with horseradish peroxidase-labeled secondary antibody at room temperature for 2 h. Finally, the protein bands were visualized by chemiluminescence, and Gel-Pro Analyzer software (Media Cybernetics, Rockville, MD, USA) was used for optical density analysis.

### 2.10. Alizarin Red Staining Assay

Treated NRK-52E cells were washed three times with PBS, fixed in 4% formaldehyde for 15 min, and washed again with PBS. The cells were then stained with 0.2% alizarin red for 5–30 min. The stained cells were observed under a microscope (BX53, Olympus) and Image-Pro Plus 6.0 software (Media Cybernetics) was used to measure the optical density of calcium salt deposits in the images.

### 2.11. Animal Experimental Design

Twenty-four six-week-old male SD rats were purchased from the Experimental Animal Research Center of Hubei (Wuhan, China) (SCXK: 2017-0012). The rats were randomly divided into four groups: a blank control group, a kidney stone model group, a treatment group, and a solvent control group. Rats in the blank control group ate and drank normally for three months. In the kidney stone model group, rats were intraperitoneally injected with 1,25(OH)_2_D_3_ (1 *μ*g/kg) every other day for three consecutive months. Rats in the treatment group were intraperitoneally injected with 1,25(OH)_2_D_3_ (1 *μ*g/kg) every other day and with MMP-9 Inhibitor I (40 *μ*g/kg) every other day for three months. In the solvent control group, rats were intraperitoneally injected with 1,25(OH)_2_D_3_ (1 *μ*g/kg) every other day and with the same amount of DMSO every other day for three consecutive months. The study was approved by the Animal Care and Use Committee of Tongji Hospital, Tongji Medical College, Huazhong University of Science and Technology. At the end of the experiment, 24 h urine and serum samples were collected from each group, and all the animals were euthanized.

### 2.12. General and Biochemical Characteristic Analysis

The weight of the rats was measured every two weeks. A Rat MMP-9 ELISA Kit was used to detect serum MMP-9 concentrations. The samples were processed and holes for the blank, standard, and sample were set on the ELISA plate. Biotinylated antibody working solution (100 *μ*l/well) was added to the ELISA plate and incubated at 37°C for 1 h, followed by enzyme-binding working solution (100 *μ*l/well, 37°C for 30 min), chromogenic agent (90 *μ*l/well, 37°C in dark for 15 min), and finally stop solution (50 *μ*l). The optical density of each hole at 450 nm was immediately detected with a spectrophotometer (Thermo Fisher Scientific). Urinary oxalate excretion levels were detected using an oxalic acid colorimetric assay kit following the manufacturer's instructions. The absorbance of each well at 450 nm was measured with a spectrophotometer (Thermo Fisher Scientific), and the urinary oxalate concentration of each group was then calculated according to the formula. Urinary calcium excretion levels were also detected using a calcium assay kit following the manufacturer's instructions. The absorbance of each well at 610 nm was measured with a spectrophotometer (Thermo Fisher Scientific), and the urinary calcium concentration of each group was then calculated according to the formula.

### 2.13. Rat Kidney Histological Analysis

Rat kidney tissue was fixed in 10% phosphate-buffered formalin solution and paraffin-embedded. The paraffin-embedded tissue was cut into 5 *μ*m thick sections. The tissue sections were then dewaxed and stained with hematoxylin and eosin to evaluate the morphology of rat kidney tissue. Apoptosis in rat kidney tissue was evaluated by the TUNEL assay. Subsequently, immunohistochemistry was used to detect protein expression levels in rat kidney tissues. Calcium crystal deposition in rat kidneys was detected by von Kossa staining. Double-standard immunofluorescence staining was used to observe the expression of RUNX2 and E-cadherin in the kidney tissue sections. Briefly, after antigen retrieval, the tissue sections were incubated overnight with anti-RUNX2 (1 : 100) and anti-E-cadherin (1 : 200) antibodies at low temperature and then with a fluorescence-labeled secondary antibody. Finally, the samples were observed and images were captured under a fluorescence microscope. All the above tissue sections were visualized using an optical microscope (BX53, Olympus) and quantitatively analyzed by ImageJ software.

### 2.14. Statistical Analysis

All experiments were performed independently at least 3 times, and all data are shown as means ± standard error of the mean(SEM). Independent-sample *t*-tests were used to compare between-group differences. One-way analysis of variance (ANOVA) with Tukey's multiple comparison test was used for comparisons among multiple groups. SPSS 25.0 (SPSS Inc., Chicago, IL, USA) was used for statistical analysis. A *P* value < 0.05 was considered statistically significant.

## 3. Results

### 3.1. A High Calcium Concentration Inhibited the Viability and Promoted the Apoptosis and Intracellular ROS Production of NRK-52E Cells

**Figure 1:**
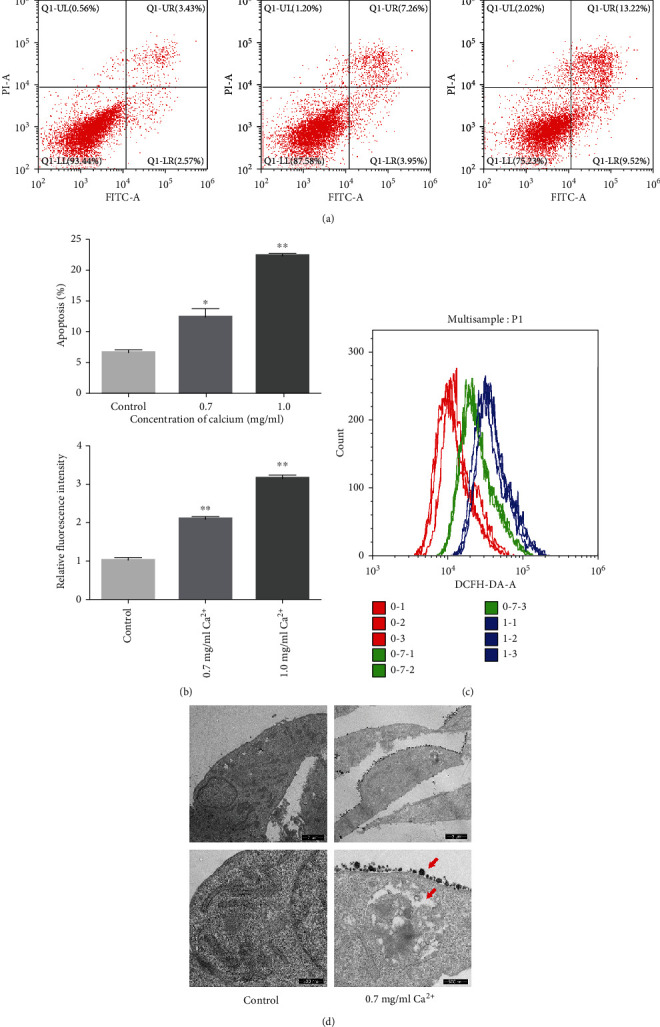
Effects of high concentration of calcium on cell apoptosis and intracellular ROS level in NRK-52E cells. (a) Flow cytometry was used to determine the proportion of apoptotic cells after treatment with high concentration of calcium for 24 hours. (b) The bar graphs showed the proportion of apoptotic cells in each group. (c) Flow cytometry was used to detect the production of ROS in cells treated with high concentration of calcium for 24 hours. The bar graphs showed the relative fluorescence intensity of each group. (d) Ultrastructural changes of cells treated with high concentration of calcium for 24 hours under TEM. The red arrows indicated the “mucinous” substances. ^∗^*P* < 0.05 versus the control group, ^∗∗^*P* < 0.01 versus the control group.

First, we preliminarily investigated the effect of a high calcium concentration on NRK-52E cells. The CCK-8 assay showed that the viability of NRK-52E cells decreased significantly when the calcium concentration reached 0.7 mg/ml (Supplementary Fig. S2). Flow cytometric analysis further showed that the apoptosis and intracellular ROS production of NRK-52E cells were significantly increased in a calcium concentration-dependent manner (Figures 1(a)–1(c)). We also measured LDH release to assess whether a high concentration of calcium would affect NRK-52E cell membrane integrity. The results showed that the LDH release level was also significantly increased in a calcium concentration-dependent manner (Supplementary Fig. S3). Accordingly, we used a calcium concentration of 0.7 mg/ml for subsequent experiments. To further explore the influence of high-calcium treatment on the ultrastructure of NRK-52E cells, we evaluated the morphological changes occurring in NRK-52E cells treated with a high calcium concentration (0.7 mg/ml) using TEM. The results showed that, compared with the control group, the surface of NRK-52E cells treated with calcium became rougher, and intracellular vacuoles and extracellular crystal adhesion could be observed (Figure 1(d)).

### 3.2. A High Calcium Concentration Promoted the Expression of MMP-9 in NRK-52E Cells and the Kidneys of SD Rats

**Figure 2:**
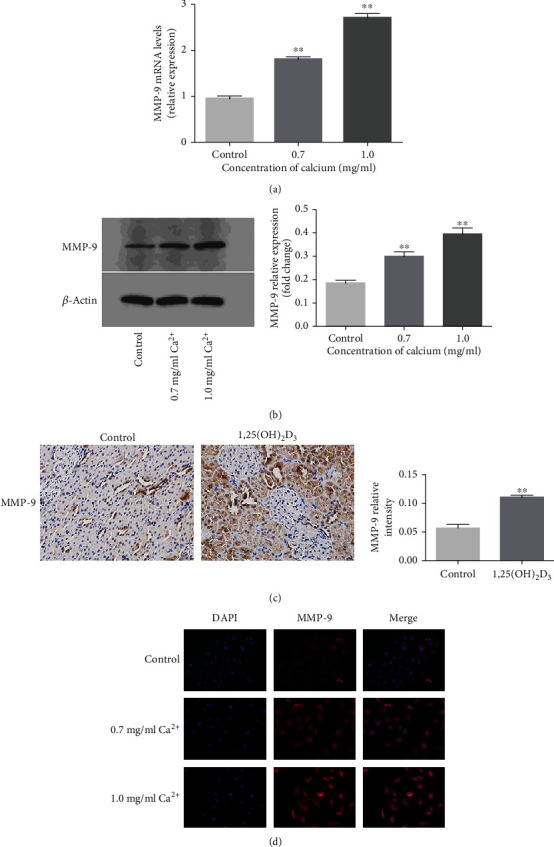
Effects of high-calcium microenvironment on the expressions of MMP-9 in NRK-52E cells and SD rat kidney. (a) The expression of MMP-9 mRNA was detected by q-PCR assay after treatment with 0.7 mg/ml and 1.0 mg/ml calcium for 24 hours. (b) The expression of MMP-9 protein was detected by western blot assay after treatment with 0.7 mg/ml and 1.0 mg/ml calcium for 24 hours. Quantitative analysis of the densities of western blot bands. (c) The expressions of MMP-9 in kidney tissues from 1,25(OH)_2_D_3_-induced rats and SD rats were detected by immunohistochemical staining (magnification 400x). Quantification of immunohistochemical staining for MMP-9 by Image-Pro Plus software. (d) The expression of MMP-9 was detected by immunofluorescence assay after treatment with 0.7 mg/ml and 1.0 mg/ml calcium for 24 hours (magnification 400x). ^∗^*P* < 0.05 versus the control group, ^∗∗^*P* < 0.01 versus the control group.

Our previous genome-wide gene expression profile analysis of GHS vs. SD rats showed that MMP-9 expression was higher in the GHS rat kidney than in that of the SD rat [10]. Here, we found that treatment with a high concentration of calcium led to a significant increase in the mRNA and protein expressions of MMP-9 in NRK-52E cells (Figures 2(a) and 2(b)). Next, immunohistochemical assays demonstrated that, compared with controls, MMP-9 expression was significantly increased in the kidneys of 1,25(OH)_2_D_3_-treated rats (Figure 2(c)). Immunofluorescence analysis showed similar effects. In the basal state, MMP-9 expression in NRK-52E cells was low and mainly concentrated in the cytoplasm; in contrast, MMP-9 expression was significantly increased and distributed in both the cytoplasm and nucleus after treatment with a high concentration of calcium (Figure 2(d)). These results suggested that MMP-9 expression was dysregulated in NRK-52E cells in a high-calcium microenvironment and may play an important role in kidney stone formation.

### 3.3. A High Calcium Concentration Promoted the Activation of the NF-*κ*B Signaling Pathway and the Expression of Osteoblastic-Related Proteins in NRK-52E Cells

**Figure 3:**
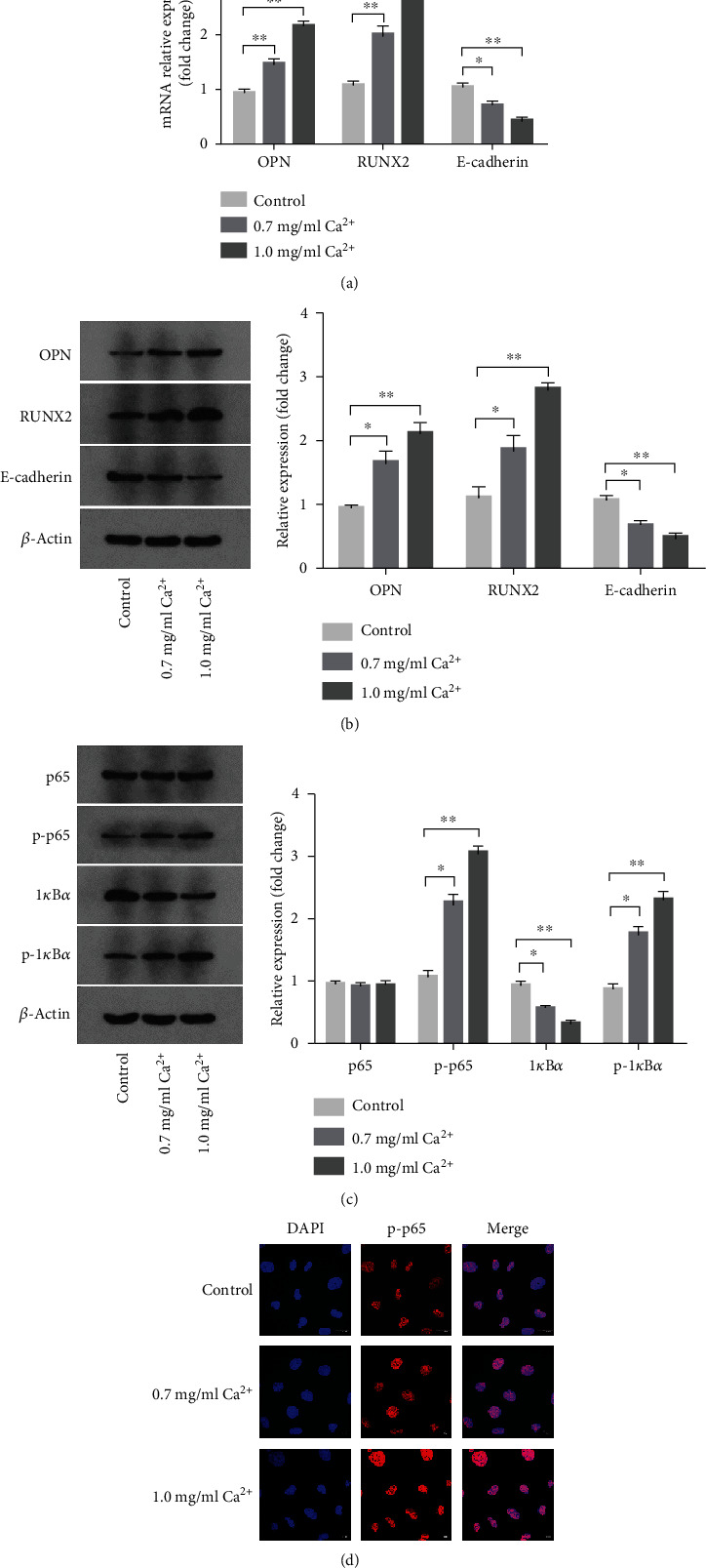
Effects of high concentration of calcium on the expressions of NF-*κ*B signaling pathway proteins and osteoblastic-related proteins in NRK-52E cells. (a) The expressions of OPN, RUNX2, and E-cadherin mRNAs were detected by q-PCR assay after treatment with 0.7 mg/ml and 1.0 mg/ml calcium for 24 hours. (b) The expressions of OPN, RUNX2, and E-cadherin proteins were detected by western blot assay after treatment with 0.7 mg/ml and 1.0 mg/ml calcium for 24 hours. Quantitative analysis of the densities of western blot bands. (c) The expressions of NF-*κ*B signaling pathway proteins were detected by western blot assay after treatment with 0.7 mg/ml and 1.0 mg/ml calcium for 24 hours. Quantitative analysis of the densities of western blot bands. (d) Intracellular p-p65 distribution was detected by immunofluorescence assay after treatment with 0.7 mg/ml and 1.0 mg/ml calcium for 24 hours (magnification 1000x). ^∗^*P* < 0.05 versus the control group, ^∗∗^*P* < 0.01 versus the control group.

We found that a high concentration of calcium significantly upregulated the expression of osteoblastic-related factors (OPN, RUNX2) at both the mRNA and protein levels and downregulated that of the epithelial marker E-cadherin in NRK-52E cells (Figures 3(a) and 3(b)). In addition, when NRK-52E cells were treated with 0.7 or 1.0 mg/ml calcium, the levels of phosphorylated p65 and I*κ*B*α* were increased, indicating that the NF-*κ*B signaling pathway was activated (Figure 3(c)). An immunofluorescence assay confirmed this effect. Compared with the control group, phosphorylated p65 expression was increased in the nuclei of NRK-52E cells after treatment with a high calcium concentration (Figure 3(d)). These results suggested that kidney stone formation in a high-calcium microenvironment was accompanied by the activation of the NF-*κ*B signaling pathway and epithelial–osteoblastic transformation in NRK-52E cells.

### 3.4. NAC Inhibited NF-*κ*B Signaling Activation and the Expression of MMP-9 and Osteoblastic-Related Proteins in NRK-52E Cells Exposed to Calcium

**Figure 4:**
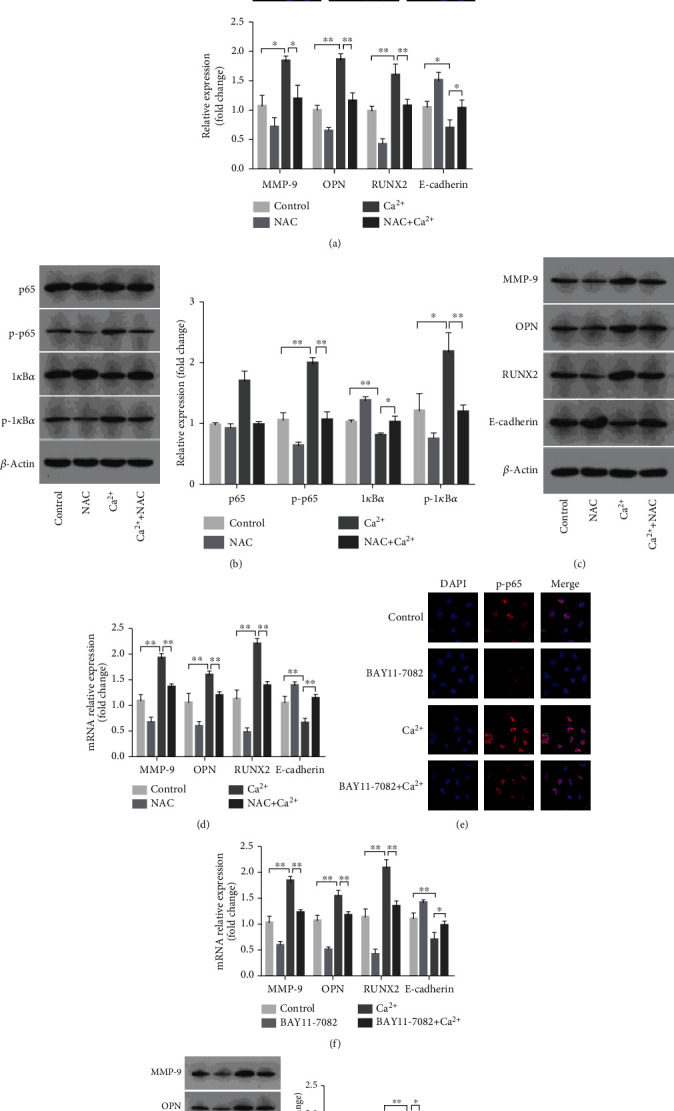
Effects of NAC and BAY 11-7082 on MMP-9 and osteoblastic-related protein expressions in NRK-52E cells exposed to calcium. (a) Intracellular p-p65 distribution was detected by immunofluorescence assay after pretreatment with or without NAC (magnification 1000x). (b) The expressions of NF-*κ*B signaling pathway proteins were detected by western blot assay in NRK-52E cells with or without NAC pretreatment. Quantitative analysis of the densities of western blot bands. (c) The expressions of MMP-9, OPN, RUNX2, and E-cadherin proteins were detected by western blot assay in NRK-52E cells with or without NAC pretreatment. Quantitative analysis of the densities of western blot bands. (d) The expressions of MMP-9, OPN, RUNX2, and E-cadherin mRNAs were detected by q-PCR assay in NRK-52E cells with or without NAC pretreatment. (e) Intracellular p-p65 distribution was detected by immunofluorescence assay after pretreatment with or without BAY 11-7082 (magnification 1000x). (f) The expressions of MMP-9, OPN, RUNX2, and E-cadherin mRNAs were detected by q-PCR assay in NRK-52E cells with or without BAY 11-7082 pretreatment. (g) The expressions of MMP-9, OPN, RUNX2, and E-cadherin proteins were detected by western blot assay in NRK-52E cells with or without BAY 11-7082 pretreatment. Quantitative analysis of the densities of western blot bands. ^∗^*P* < 0.05 versus the control group, ^∗∗^*P* < 0.01 versus the control group.

Increased ROS generation has been reported to activate the NF-*κ*B signaling pathway, while MMP-9 is a known downstream target of NF-*κ*B [9, 10]. Here, our results showed consistent changes in the levels of ROS, NF-*κ*B signaling pathway-related proteins, MMP-9, and osteoblastic-related proteins. This suggested that there might be a connection between these molecules during kidney stone formation. We first explored the role of ROS in this process. Immunofluorescence results showed that, in calcium-exposed NRK-52E cells pretreated with the ROS scavenger NAC, the level of phosphorylated p65 in the nucleus was significantly reduced (Figure 4(a)). Western blot results also showed significantly reduced levels of phosphorylated p65 and I*κ*B*α*, indicating that the NF-*κ*B signaling pathway was inhibited (Figure 4(b)). We also found that the mRNA and protein expression levels of MMP-9 and those of the osteoblastic-related proteins OPN and RUNX2 were decreased, whereas those of the epithelial marker E-cadherin were increased, in NRK-52E cells after pretreatment with NAC (Figures 4(c) and 4(d)). These results suggested that excessive ROS production in NRK-52E cells under a high-calcium microenvironment influenced calcium crystal deposition by regulating the NF-*κ*B signaling pathway and MMP-9 expression.

### 3.5. BAY 11-7082 Inhibited the Expression of MMP-9 and That of Osteoblastic-Related Proteins in NRK-52E Cells Exposed to Calcium

To further explore the role of the NF-*κ*B signaling pathway in kidney stone formation, calcium-treated NRK-52E cells were further exposed to BAY 11-7082, an inhibitor of I*κ*B*α* phosphorylation. Immunofluorescence staining showed that phosphorylated p65 expression was decreased in the nuclei of the NRK-52E cells treated with BAY 11-7082, indicating that the NF-*κ*B signaling pathway was inhibited (Figure 4(e)). We also found that the expression of MMP-9 and that of the osteoblastic-related proteins OPN and RUNX2 was decreased, whereas that of the epithelial marker E-cadherin was increased, at both the mRNA and protein levels in NRK-52E cells after pretreatment with BAY 11-7082 (Figures 4(f) and 4(g)). These results indicated that the NF-*κ*B signaling pathway activated by increased ROS production in NRK-52E cells under a high-calcium microenvironment influenced calcium crystal deposition by regulating the expression of its downstream target, MMP-9.

### 3.6. The Knockdown or Overexpression of MMP-9 Affected Epithelial–Osteoblastic Transformation and Calcium Crystal Deposition in NRK-52E Cells

**Figure 5:**
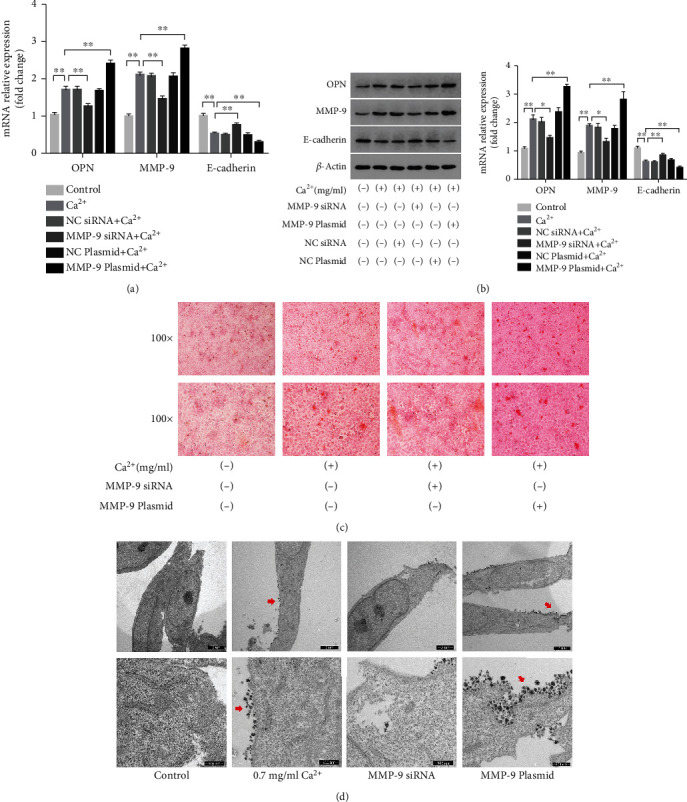
Knockdown or overexpression of MMP-9 affected epithelial–osteoblastic transformation and calcium crystal deposition of NRK-52E cells. (a) The expressions of OPN, RUNX2, and E-cadherin mRNAs were detected by q-PCR assay in NRK-52E cells with or without high-calcium exposure, as well as MMP-9 knockdown or overexpression. (b) The expressions of OPN, RUNX2, and E-cadherin proteins were detected by western blot assay in NRK-52E cells with or without high-calcium exposure, as well as MMP-9 knockdown or overexpression. Quantitative analysis of the densities of western blot bands. (c) Calcium crystal deposition was detected by Alizarin red staining in NRK-52E cells with or without high-calcium exposure, as well as MMP-9 knockdown or overexpression. (d) Ultrastructure was observed by TEM in NRK-52E cells with or without high-calcium exposure, as well as MMP-9 knockdown or overexpression. The red arrows indicated the crystal adhesion. ^∗^*P* < 0.05 versus the control group, ^∗∗^*P* < 0.01 versus the control group.

Given that MMP-9 expression was dysregulated in NRK-52E cells exposed to calcium, and that this effect was mediated by ROS levels and the NF-*κ*B signaling pathway, we next performed an MMP-9 loss- and gain-of-function analysis using MMP-9 siRNA and an MMP-9 expression plasmid, respectively, to explore the role of this protein in kidney stone formation. In NRK-52E cells treated with or without a high concentration of calcium, the expression of the osteoblastic-related proteins OPN and RUNX2 was decreased, whereas that of the epithelial marker E-cadherin was increased, at both the mRNA and protein levels in MMP-9-silenced cells; meanwhile, MMP-9 overexpression elicited the opposite effect (Figures 5(a) and 5(b)). Subsequently, alizarin red staining of NRKK-52E cells treated with a high calcium concentration (0.7 mg/ml) indicated that calcium crystal deposition was lower in the MMP-9-silenced group than in the control group, whereas the opposite result was obtained when MMP-9 was overexpressed (Figure 5(c)). TEM analysis demonstrated that the surface of NRK-52E cells in the MMP-9 siRNA-treated group was relatively smooth, while intracellular vacuole formation and extracellular crystal adhesion were reduced or absent compared with those in the calcium-only treatment group (Figure 5(d)). These results suggested that the abnormal expression of MMP-9 in NRK-52E cells under a high-calcium microenvironment affected calcium crystal deposition, i.e., high MMP-9 expression promoted crystal deposition, whereas low MMP-9 expression inhibited this process.

### 3.7. The Effects of MMP-9 Inhibitor I on the Pathophysiological and Morphological Changes Induced by 1,25(OH)_2_D_3_ in SD Rats

**Figure 6:**
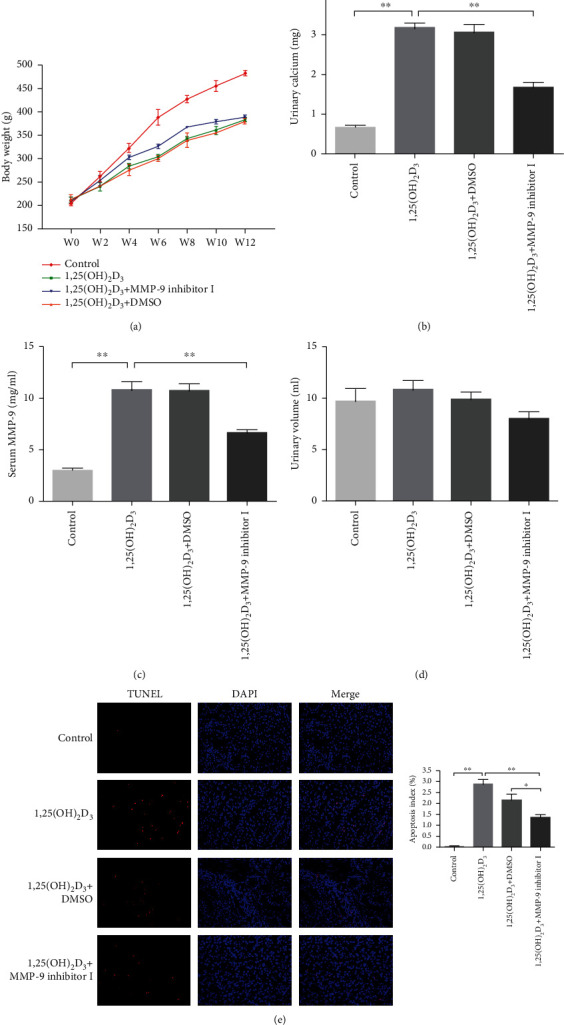
Effects of MMP-9 inhibitor I on pathophysiological and morphological changes induced by 1,25(OH)_2_D_3_ in SD rats. (a) Body weight changes in each group every two weeks. (b) Urinary calcium excretion levels of rats in each group at the end of the experiment. (c) Serum MMP-9 concentrations of rats in each group at the end of the experiment. (d) 24-hour urine volumes of rats in each group at the end of the experiment. (e) The apoptotic levels of renal tubules in each group were detected by TUNEL staining assay. Apoptotic index between groups. ^∗^*P* < 0.05 versus the control group, ^∗∗^*P* < 0.01 versus the control group.

1,25(OH)_2_D_3_ was used to generate a rat model of hypercalciuria. First, we assessed whether treatment with a high calcium concentration would elicit pathophysiological and/or morphological changes in rat kidneys. At the end of the animal experiment, there was no statistically significant difference in the intervention-related mortality among the groups. There was also no significant difference in body weight among the three treatment groups; however, the body weight of the rats in all the treatment groups was significantly lower than that of rats in the control group (Figure 6(a)). Urinary calcium excretion levels and serum MMP-9 concentrations in the 1,25(OH)_2_D_3_ and 1,25(OH)_2_D_3_+DMSO treatment groups were significantly higher than those of the control group; however, the administration of MMP-9 inhibitor I significantly reduced urinary calcium excretion levels (Figure 6(b), Supplementary Table S6) and serum MMP-9 concentrations in the animals (Figure 6(c)). There was no significant difference in 24 h urine volumes among the groups (Figure 6(d)). TUNEL staining showed that the 1,25(OH)_2_D_3_ and 1,25(OH)_2_D_3_+DMSO groups displayed a greater number of apoptotic cells compared with the control group, while MMP-9 inhibitor I treatment led to a significant reduction in apoptosis levels (Figure 6(e)).

### 3.8. MMP-9 Inhibitor I Attenuated Calcium Crystal Deposition and Osteoblastic-Related Protein Expression Induced by 1,25(OH)_2_D_3_ in SD Rat Kidneys

**Figure 7:**
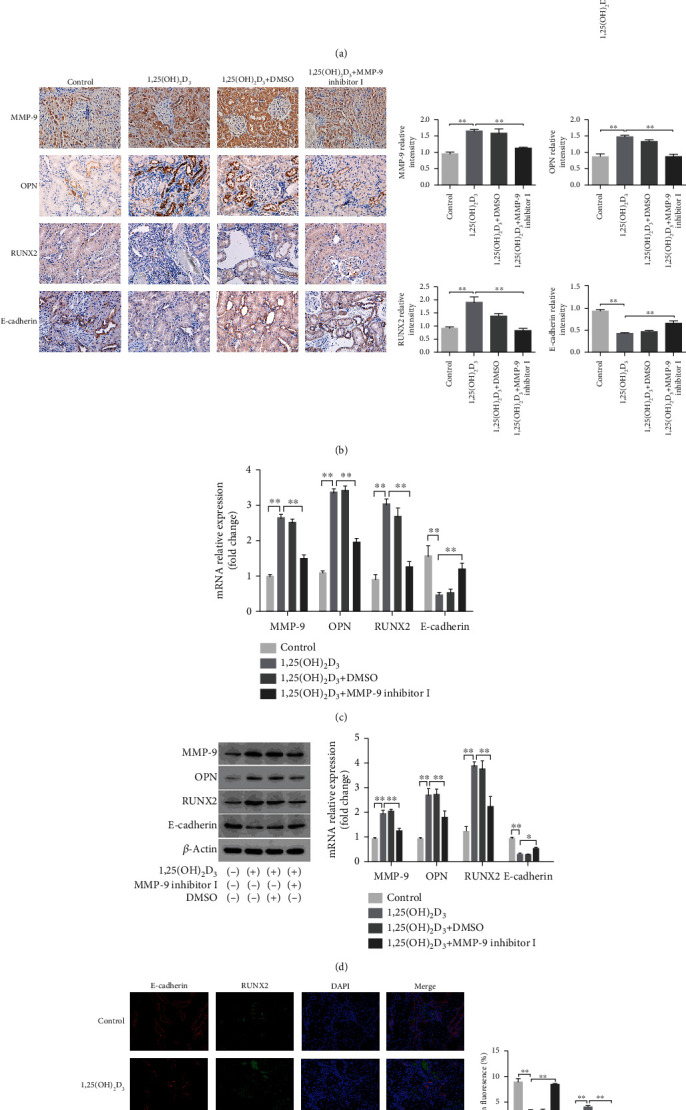
Effects of MMP-9 inhibitor I on calcium crystal deposition and osteogenic related proteins expression in 1,25(OH)_2_D_3_-induced SD rat kidney. (a) von Kossa staining detected calcium crystal deposition at the junction of renal cortex and medulla in 1,25(OH)2D3-induced SD rat kidney (Aa–d, magnification 100x; Ae–h, magnification 200x). Quantification of calcium crystal deposition level. (b) The expressions of MMP-9, OPN, RUNX2, and E-cadherin in rat kidney tissues were detected by immunohistochemical staining (magnification 400x). Quantification of immunohistochemical staining for MMP-9, OPN, RUNX2, and E-cadherin by Image-Pro Plus software. (c) The expressions of MMP-9, OPN, RUNX2, and E-cadherin mRNAs were detected by q-PCR assay in rat kidney tissues. (d) The expressions of MMP-9, OPN, RUNX2, and E-cadherin proteins were detected by western blot assay in rat kidney tissues. Quantitative analysis of the densities of western blot bands. (e) Immunofluorescence double-standard staining was used to detect the expression differences of RUNX2 and E-cadherin. Quantification of the mean fluorescence values of RUNX2 and E-cadherin in rat renal tissue. ^∗^*P* < 0.05 versus the control group, ^∗∗^*P* < 0.01 versus the control group.

The von Kossa staining method was used to detect calcium crystal deposition at the boundary between the cortex and medulla of the rat kidney. The results showed that calcium crystal deposition was similar between the 1,25(OH)_2_D_3_ and 1,25(OH)_2_D_3_+DMSO groups; however, deposition was significantly higher in both treatment groups than in the control group. After treatment with MMP-9 inhibitor I, calcium crystal deposition was significantly reduced in both the 1,25(OH)_2_D_3_ and 1,25(OH)_2_D_3_+DMSO groups (Figure 7(a)). Immunohistochemistry results demonstrated that compared with controls, the protein expression levels of MMP-9, OPN, and RUNX2 were significantly decreased, while that of E-cadherin was increased, in the kidneys of rats treated with MMP-9 inhibitor I (Figure 7(b)). Real-time quantitative polymerase chain reaction and western blot analysis showed similar results (Figures 7(c) and 7(d)). Double-standard immunofluorescence staining analysis showed that the fluorescence intensity of RUNX2 (green) was increased and that of E-cadherin decreased (red) in the 1,25(OH)_2_D_3_ and 1,25(OH)_2_D_3_+DMSO groups compared with that of the control group, indicating that epithelial–osteoblastic transformation had occurred in renal tubular cells. However, after treatment with MMP-9 inhibitor I, the RUNX2 fluorescence intensity was reduced and that of E-cadherin increased compared with that in the 1,25(OH)_2_D_3_ and 1,25(OH)_2_D_3_+DMSO groups (Figure 7(e)). These results suggested that the inhibition of MMP-9 expression can attenuate hypercalciuria-induced epithelial–osteoblastic transformation and calcium crystal deposition.

## 4. Discussion

In the current study, we found that stimulation with a high concentration of calcium can promote the expression of MMP-9 in renal tubular epithelial cells. Silencing MMP-9 gene expression slowed the transformation from an epithelial phenotype to an osteoblast-like phenotype, inhibited the expression of the osteogenesis-related protein OPN, and inhibited calcium salt deposition, whereas the opposite effects were observed when MMP-9 was overexpressed. We have previously shown that exposure to a high calcium concentration can activate the NF-*κ*B signaling pathway by promoting the production and release of intracellular ROS, which, in turn, activated the expression of MMP-9, a target of NF-*κ*B, thereby leading to kidney stone formation. The results of the present study further contribute to elucidating the mechanisms involved in kidney stone formation.

The kidney stone disease is a chronic systemic condition with increasing prevalence worldwide and is associated with obesity, diabetes, metabolic syndrome, and other increasingly common diseases [17, 18]. Several studies have linked idiopathic kidney stone formation to vascular calcification, a process similar to bone formation [19–23]. It has been reported that MMPs, in addition to their cannonical function in degrading extracellular matrix molecules [24], also have a nonmatrix-associated role in osteogenesis and are involved in several aspects of mesenchymal stem cell renewal and differentiation [25, 26]. Vu et al. [27] found defects in endochondral ossification in MMP-9-deficient mice. Colnot et al. [28] also found that MMP-9 expression was increased during bone healing after fracture in mice, while fracture healing was slower in mice lacking MMP-9 than in normal mice. Moreover, several studies have reported that MMP-9 plays an important role in vascular calcification [11–15]. However, the role of MMP-9 in kidney stone formation is unclear. We previously reported that MMP-9 expression was abnormally elevated in the kidney tissues of GHS rats compared with that in normal SD rats [10]. To explore the role of MMP-9 in high calcium concentration-induced kidney stone formation, we first examined the effect of exposure to a high concentration of calcium on the expression of MMP-9 in NRK-52 cells. We found that calcium stimulation could promote the expression of MMP-9 in a concentration-dependent manner, accompanied by the upregulation of the osteogenesis-related protein OPN and the osteogenic marker RUNX2, as well as the downregulation of the epithelial marker E-cadherin. These results suggested that MMP-9 is involved in kidney stone formation.

To further explore whether MMP-9 plays a lithogenic or inhibitory role in kidney stone formation, we knocked down or overexpressed MMP-9 in NRK-52E cells. The results showed that silencing MMP-9 suppressed the expression of the osteogenesis-related protein OPN and that of the osteogenic marker RUNX2 and inhibited mineralization, while also promoting the expression of the epithelial marker E-cadherin; in contrast, MMP-9 overexpression elicited the opposite effects. These findings indicate that MMP-9 plays an important catalytic role in kidney stone formation. However, the specific mechanism by which MMP-9 promotes kidney stone formation may be complex and warrant further exploration. MMPs can act as release agents for bioactive fragments of extracellular matrix molecules and influence cell behavior through these effects [29]. Ortega et al. [25] found that MMPs have a nonmatrix role during osteogenesis, while Mannello et al. [26] reported that they are also involved in the regeneration and differentiation of mesenchymal stem cells. Our data suggest that MMP-9 may promote the osteoblastic transformation of NRK-52E cells, leading to calcium salt deposition. Our results further demonstrated that MMP-9 can promote the adhesion of crystals to the cell surface. Recent studies have suggested that MMPs may be involved in the regulation of inflammation [30]. In addition to acting as downstream regulators of inflammatory cytokines, MMPs can also significantly influence matrix biology by exposing stromal fragments (e.g., elastin peptides) and new collagen epitopes, and these can subsequently act as signaling molecules that control angiogenesis and inflammation [29]. These results indicate that MMPs themselves may play a proinflammatory role in vascular calcification [31]. Combined, these observations suggest that MMP-9 may promote stone formation by regulating the inflammatory response. In addition, microscopic analysis by Khan et al. [32] showed that apatite deposition in the interstitium is closely related to cellular degradation products, membrane-bound vesicles, and several fibrous substances and collagen fibers. The authors suggested that Randall's plaques are formed through collagen mineralization, and the mineralized “front” expands to the renal papillary epithelium. This implies that MMP-9 may promote Randall's plaque formation and, consequently, kidney stone formation, by promoting the transfer of crystalline particles to the renal interstitium. Moreover, MMP-9 may cause the surface of Randall's plaques to rupture, allowing contact between the hydroxyapatite and pelvic urine, thereby forming the core of calcium salt deposition that leads to kidney stone formation [33, 34].

Oxidative stress is known to be involved in the pathogenesis of many diseases. A large number of clinical and experimental studies have confirmed that ROS may be involved in the pathogenesis of idiopathic calculi [35–37]. Gambaro et al. [21] found that renal tubular epithelial cells under oxidative stress may become osteoblastic-like vascular smooth muscle cells during vascular calcification. In addition, studies have shown that ROS can activate the NF-*κ*B signaling pathway [9]. We previously also found that NF-*κ*B signaling is a key component of the gene regulatory network in GHS rats and that MMP-9 is a downstream target of NF-*κ*B [10]. Accordingly, we observed that intracellular ROS levels were increased after treatment with different concentrations of calcium. This was similar to the results of Li et al. [38] who reported that a high calcium concentration stimulates ROS production in the respiratory chain; this increase activates the calcium channel in the endoplasmic reticulum, leading to the release of more Ca^2+^ and thereby further promoting ROS release and increased the intracellular phosphorylation of p65 and I*κ*B*α*, indicating that the NF-*κ*B pathway was activated. Then, we pretreated cells with the ROS scavenging agent NAC and the NF-*κ*B inhibitor BAY 11-7082 and found that the expression of MMP-9, OPN (an osteogenesis-related protein), and RUNX2 (an osteogenic marker) was inhibited. These results suggest that a high concentration of calcium promotes MMP-9 expression and kidney stone formation through ROS and NF-*κ*B signaling pathways.

We also used a vitamin D-induced hypercalciuria rat model to verify the regulatory role of MMP-9 in high calcium concentration-induced kidney stone formation. Wang et al. [39] reported that the commonly used aldehyde-induced kidney stone rat model simulates a hyperoxaluric microenvironment in which crystals assemble and plug in the terminal manifold, much like Randall's plugs. However, rats in the vitamin D-induced model developed a hypercalciuric microenvironment, and the crystals initially formed in the basement membrane of the loop of Henle, extending into the vasa recta and interstitial tissue, which was more like that seen for Randall's plaques [40]. Here, the results showed that the expression of MMP-9, OPN, and RUNX2 was significantly increased, as was the mineralization of renal tissue, in our model rats, while MMP-9 inhibitor I administration elicited the opposite effects. These results were consistent with the NRKK-52E cell data, confirming that a high concentration of calcium can promote kidney stone formation by upregulating MMP-9 expression.

Finally, this study had limitations. Our results suggested that MMP-9 may promote osteogenic transformation and crystal adhesion in renal tubular epithelial cells and thus promote calcium crystal deposition. However, we did not thoroughly explore how MMP-9 promotes the deposition of calcium crystals. Studies have shown that MMP-9 may also induce calcium crystal deposition by regulating the inflammatory response, promoting the transfer of crystal particles to the renal interstitium, and promoting the surface rupture of Randall's plaques. Additionally, whether MMP-9 also has a similar role in humans remains to be determined.

## 5. Conclusions

**Figure 8:**
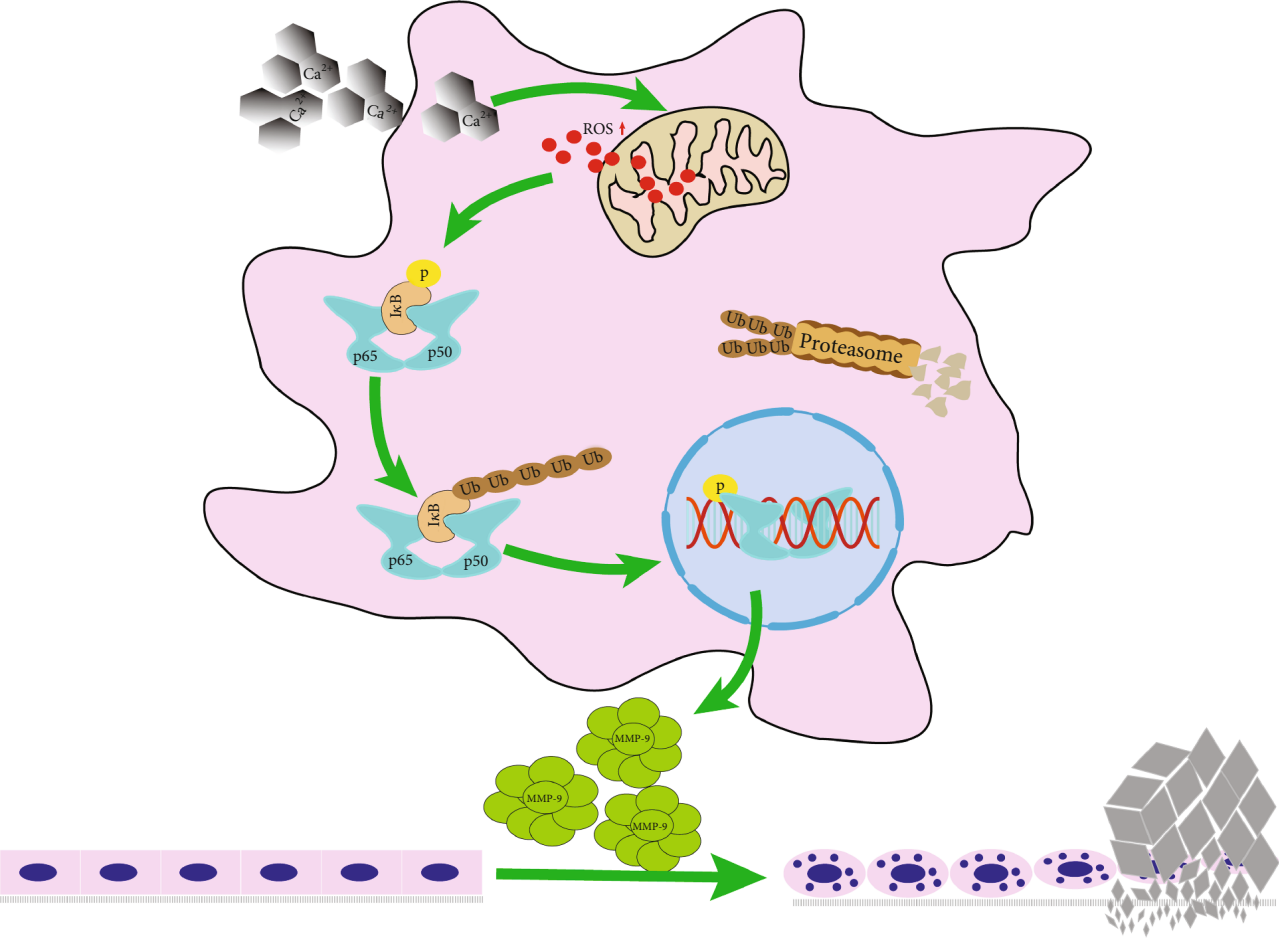
Graphical abstract.

In conclusion, our results demonstrated for the first time that a high concentration of calcium stimulates ROS production, activates the NF-*κ*B pathway, and induces the upregulation of MMP-9 expression, which promotes calcium crystal deposition in renal tubular epithelial cells both in vitro and in vivo (Figure 8). Our study revealed a novel role for MMP-9 in calcium crystal deposition and kidney stone formation induced by a high calcium concentration.

## Data Availability

The data and materials can be obtained by contacting the corresponding author.

## Abbreviations

NRK-52E: Normal rat kidney epithelial-like cell
ROS: Reaction oxygen species
NF-*κ*B: Nuclear factor kappa-light-chain-enhancer of activated B cells
MMP-9: Matrix metalloproteinase-9
SD: Sprague–Dawley
GHS: Genetic hypercalciuric stone-forming
DMEM: Dulbecco's modified Eagle medium
PBS: Phosphate-buffered saline
FBS: Fetal bovine serum
DMSO: Dimethyl sulfoxide
BSA: Bovine serum albumin
NAC: N-Acetylcysteine
CCK-8: Cell counting kit-8
DCFH-DA: 2′,7′-Dichlorofluorescein diacetate
TEM: Transmission electron microscope
SDS-PAGE: Sodium dodecyl sulfate-polyacrylamide gel electrophoresis
PVDF: Polyvinylidene difluoride
siRNA: Small interfering RNA
TUNEL: Terminal deoxynucleotidyl transferase-mediated dUTP-biotin nick end-labeling assay
RUNX2: Runt-related transcription factor 2
OPN: Osteopontin.

## Supplementary Materials

Supplementary Materials Table S1: the sequences of primer RNA. Figure S2: effects of different concentrations of calcium on the cell viability of NRK-52E cells were assessed by performing CCK-8 assay. ^∗∗^*P* < 0.01 versus the control group. Figure S3: effects of different concentrations of calcium on the LDH release levels of NRK-52E cells were assessed by LDH assay kit. ^∗∗^*P* < 0.01 versus the control group. Figure S4: transcript levels for MMP-9 were assessed following transfection of scrambled control RNA (NC siRNA) or MMP-9-specific siRNA (MMP-9 siRNA1-3) in NRK-52E. ^∗∗^*P* < 0.01 versus the control group. Figure S5: transcript levels for MMP-9 were assessed following transfection of scrambled control RNA (NC plasmid) or MMP-9 plasmid in NRK-52E. ^∗∗^*P* < 0.01 versus the control group. Table S6: the 24 h urinary calcium excretion level and concentration of rats in each group.
