# Supplementary material for: The Activation of ROS/NF-κB/MMP-9 Pathway Promotes Calcium-Induced Kidney Crystal Deposition
Source: Oxid Med Cell Longev. 2021 Jun 8;2021:8836355. doi: 10.1155/2021/8836355 (PMC8208877; doi:10.1155/2021/8836355)
Supplement: Supplementary Materials — Table S1: the sequences of primer RNA. Figure S2: effects of different concentrations of calcium on the cell viability of NRK-52E cells were assessed by performing CCK-8 assay. ∗∗P < 0.01 versus the control group. Figure S3: effects of different concentrations of calcium on the LDH release levels of NRK-52E cells were assessed by LDH assay kit. ∗∗P < 0.01 versus the control group. Figure S4: transcript levels for MMP-9 were assessed following transfection of scrambled control RNA (NC siRNA) or MMP-9-specific siRNA (MMP-9 siRNA1-3) in NRK-52E. ∗∗P < 0.01 versus the control group. Figure S5: transcript levels for MMP-9 were assessed following transfection of scrambled control RNA (NC plasmid) or MMP-9 plasmid in NRK-52E. ∗∗P < 0.01 versus the control group. Table S6: the 24 h urinary calcium excretion level and concentration of rats in each group. [file 8836355.f1.docx]

**The activation of ROS/NFκB/MMP-9 pathway promotes calcium-induced kidney crystal deposition**

| Name | Primer | Sequence | Size |
| --- | --- | --- | --- |
| Rat β-actin | Forward | 5‘- CACGATGGAGGGGCCGGACTCATC -3’ | 240bp |
|  | Reverse | 5‘- TAAAGACCTCTATGCCAACACAGT-3’ |  |
| Rat MMP-9 | Forward | 5‘-GCTGGGCTTAGATCATTCTTCAGTG-3’ | 109bp |
|  | Reverse | 5‘-CAGATGCTGGATGCCTTTTATGTCG-3’ |  |
| Rat E-cadherin | Forward | 5‘-TCGCCTACACCATCCTCAGCCAAGA-3’ | 282bp |
|  | Reverse | 5‘-CAATACGGGCACCGACCTCATTCTC-3’ |  |
| Rat OPN | Forward | 5‘-GCAGTGGTTTGCTTTTGCCTGTTCG-3’ | 237bp |
|  | Reverse | 5‘-ATGGCTTTCATTGGAGTTGCTTGGA-3’ |  |
| Rat RUNX2 | Forward | 5‘-TCCCAGTATGAGAGTAGGTGTCCCG-3’ | 296bp |
|  | Reverse | 5‘-GCCAGAGGCAGAAGTCAGAGGTG-3’ |  |

Supplementary Table S1. The sequences of primer RNA


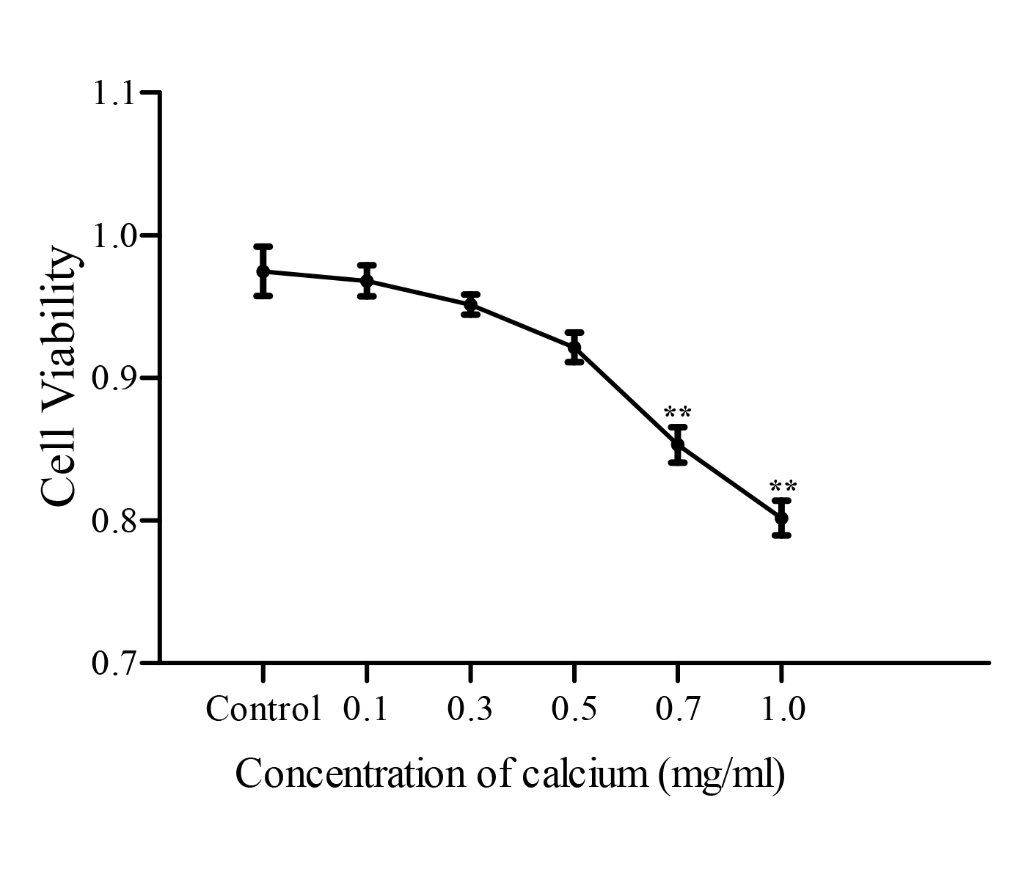


Supplementary Figure S2. Effects of different concentrations of calcium on the cell viability of NRK-52E cells were assessed by performing CCK-8 assay. ***P* < 0.01 versus control group.


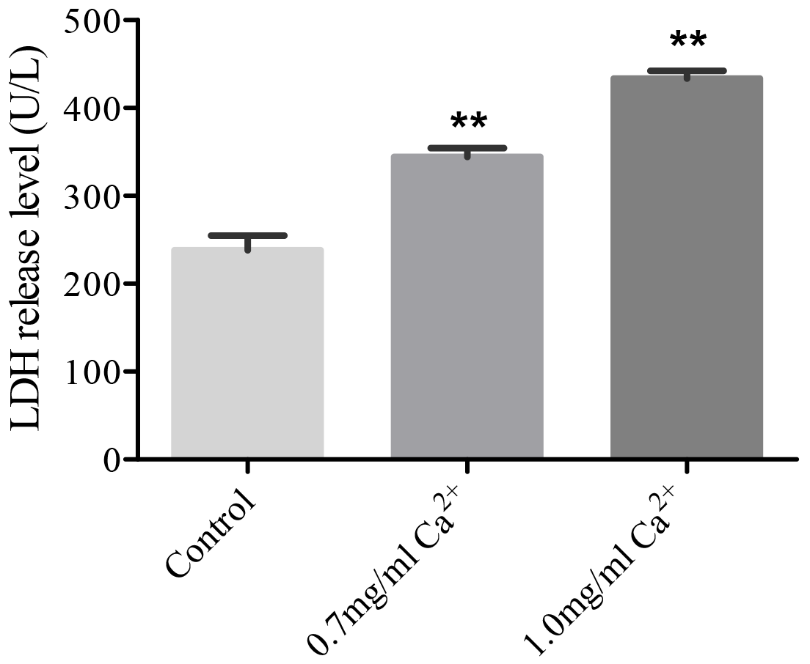


Supplementary Figure S3. Effects of different concentrations of calcium on the LDH release levels of NRK-52E cells were assessed by LDH assay kit. ***P* < 0.01 versus control group.


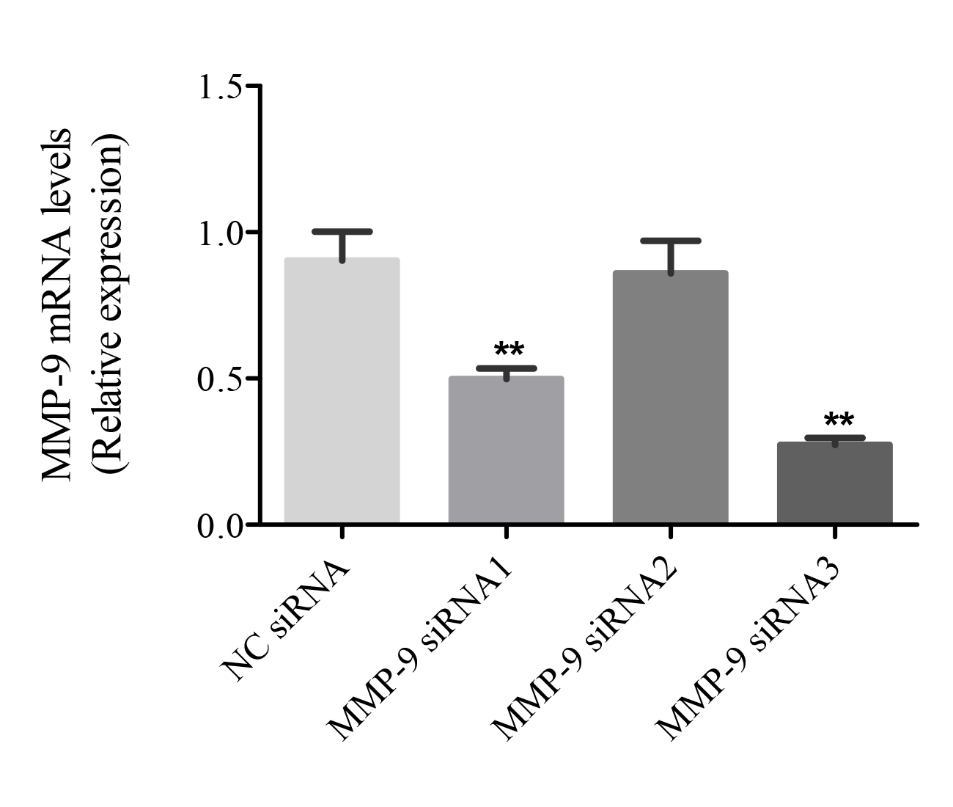


Supplementary Figure S4. Transcript levels for MMP-9 were assessed following transfection of scrambled control RNA (NC siRNA) or MMP-9-specific siRNA (MMP-9 siRNA1-3) in NRK-52E. ***P* < 0.01 versus control group.

\


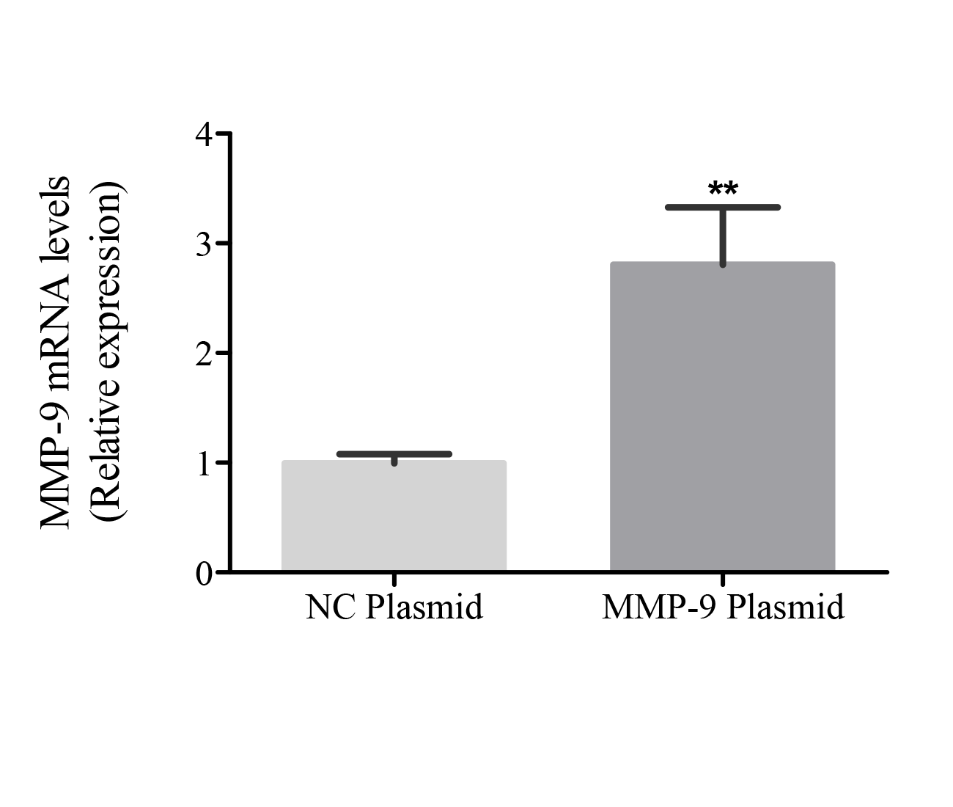


Supplementary Figure S5. Transcript levels for MMP-9 were assessed following transfection of scrambled control RNA (NC Plasmid) or MMP-9 Plasmid in NRK-52E. ***P* < 0.01 versus control group.

| Group | The 24h urinary calcium concentration (mg/ml) | The 24h urinary calcium level (mg) |
| --- | --- | --- |
| Control | 0.067±0.004 | 0.647±0.097 |
| 1,25(OH)_2_D_3_ | 0.301±0.046 | 3.202±0.135 |
| 1,25(OH)_2_D_3_+DMSO | 0.314±0.053 | 3.061±0.295 |
| 1,25(OH)_2_D_3_+MMP-9 Inhibitor Ⅰ | 0.209±0.006 | 1.666±0.169 |

Supplementary Table S6. The 24h urinary calcium excretion level and concentration of rats in each group.
